# Management Options of Giant Colonic Lipomas

**DOI:** 10.7759/cureus.23370

**Published:** 2022-03-21

**Authors:** Christopher Prien, Alya Riaz, Elie Sutton, Danny Sherwinter, Rebecca J Rhee

**Affiliations:** 1 General Surgery, Maimonides Medical Center, Brooklyn, USA; 2 Colorectal Surgery, Maimonides Medical Center, Brooklyn, USA; 3 Minimally Invasive & Bariatric Surgery, Maimonides Medical Center, Brooklyn, USA

**Keywords:** endoscopy intervention, colectomy, minimally invasive surgery, colonic intussusception, giant colonic lipoma

## Abstract

Colonic lipomas are rare, benign neoplasms, typically asymptomatic, and predominantly found incidentally during autopsy or routine surveillance. Symptomatic lesions are usually those greater than 2 cm in diameter while “giant” lesions are characterized as those over 4 cm. Presentations can vary from asymptomatic to more severe sequelae, including obstruction, gastrointestinal bleeding, or intussusception. Resection of these lesions has historically been restricted to large or symptomatic lesions. However, recent reports suggest lipomas may retain the ability to grow and can become symptomatic over time despite being inconsequential initially. This series provides a review of the clinical manifestations of colonic lipomas, radiographic characteristics, and a treatment recommendation for management of these lesions using minimally invasive surgical techniques whilst advocating for consideration of resection prior to the development of symptoms or more emergent complications.

## Introduction

Colonic lipomas are rare, benign tumors composed of adipose tissue, with a peak prevalence of 4.4% [1], second only to adenomatous polyps. Although the majority remain asymptomatic, those characterized as “giant,” or greater than 4 cm in diameter, become symptomatic in 75% of cases [2]. Symptomatology, largely correlated with size, can range from nonspecific complaints to serious complications, including hemorrhage, obstruction, or intussusception [3]. Intussusception historically has been well-described in children with occurrences in adults representing only 5% of cases [4] and usually the result of malignant lesions [5]. As the management of a colonic lipoma with resultant colocolonic intussusception merits surgical intervention, it is imperative that accurate identification and diagnosis of the underlying etiology be made [6]. However, the management of colonic lipomas in non-emergent scenarios remains a topic of discussion as advancements in endoscopic and surgical techniques have been made and intervention on asymptomatic lipomas remains a topic of debate [7-11]. In this report, we present our institution’s experience managing giant colonic lipomas with a variety of presentations and associated intussusception whilst also reviewing current management approaches in the literature.

## Case presentation

Case 1

A 71-year-old male with a history significant for benign prostatic hypertrophy and nephrolithiasis was referred for surgical evaluation after the identification of a giant colonic lipoma causing colocolonic intussusception of the sigmoid colon. The patient reported the recent development of colicky abdominal pain, bloating, and constipation, which prompted him to seek evaluation by his gastroenterologist. Previous colonoscopies had identified a colonic lipoma, but he never experienced symptoms. A review of systems revealed no pertinent findings, and he denied any family history of colon cancer. On examination, the patient’s abdomen was soft, non-tender, and non-distended. Prior to evaluation, an outpatient computed tomography (CT) scan was obtained, demonstrating a large, pedunculated mass in the sigmoid colon with possible intussusception. An urgent colonoscopy was performed, which demonstrated an ulcerated, pedunculated lesion with the head measuring 8 cm in diameter, neck 10 cm long, and the base occupying roughly 75% of the luminal circumference. No evidence of intussusception was observed endoscopically. Multiple attempts were made to resect the mass using an endoloop, but they were unsuccessful, as it was not possible to pass the endoloop over the head of the mass.

**Figure 1:**
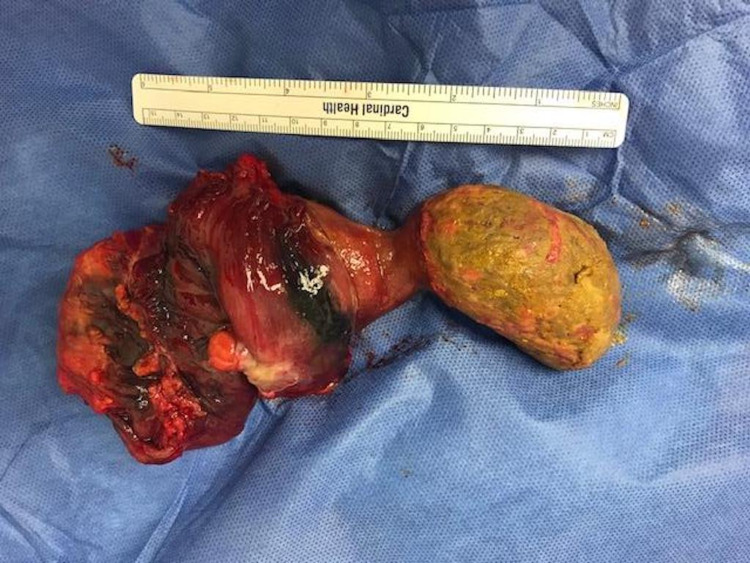
Surgical specimen demonstrating a giant, pedunculated colonic lipoma with neighboring colonic tissue grossly normal in appearance

In the setting of persistent symptoms, failed attempts at endoscopic resection, and concern for intussusception, the patient was taken for operative exploration. Intraoperatively, an area of intussusception was identified near the splenic flexure. With the diagnosis of colonic lipoma confirmed preoperatively, the decision was made to proceed with a limited resection of the mass and associated intussuscepted colon. A successful laparoscopic, segmental colectomy of the descending colon was performed with the creation of a stapled, isoperistaltic, side-to-side colocolonic anastomosis. Postoperatively, he recovered as expected and was successfully discharged on postoperative Day 2 following tolerance of an oral diet, oral analgesic medication, and return of bowel function. A pathologic review of the specimen identified a tan-yellow, ulcerated, partially infarcted, pedunculated submucosal lipoma. The head measured 6.8 x 4.7 x 4.5 cm and the stalk measured 6.0 x 3.6 x 3.0 cm with evidence of intussusception of the neighboring colonic tissue as well as 14 reactive lymph nodes (Figure 1). There was no evidence of perforation, invasion into the colonic wall, or extension of the mass to the peritonealized surface.

Case 2

**Figure 2:**
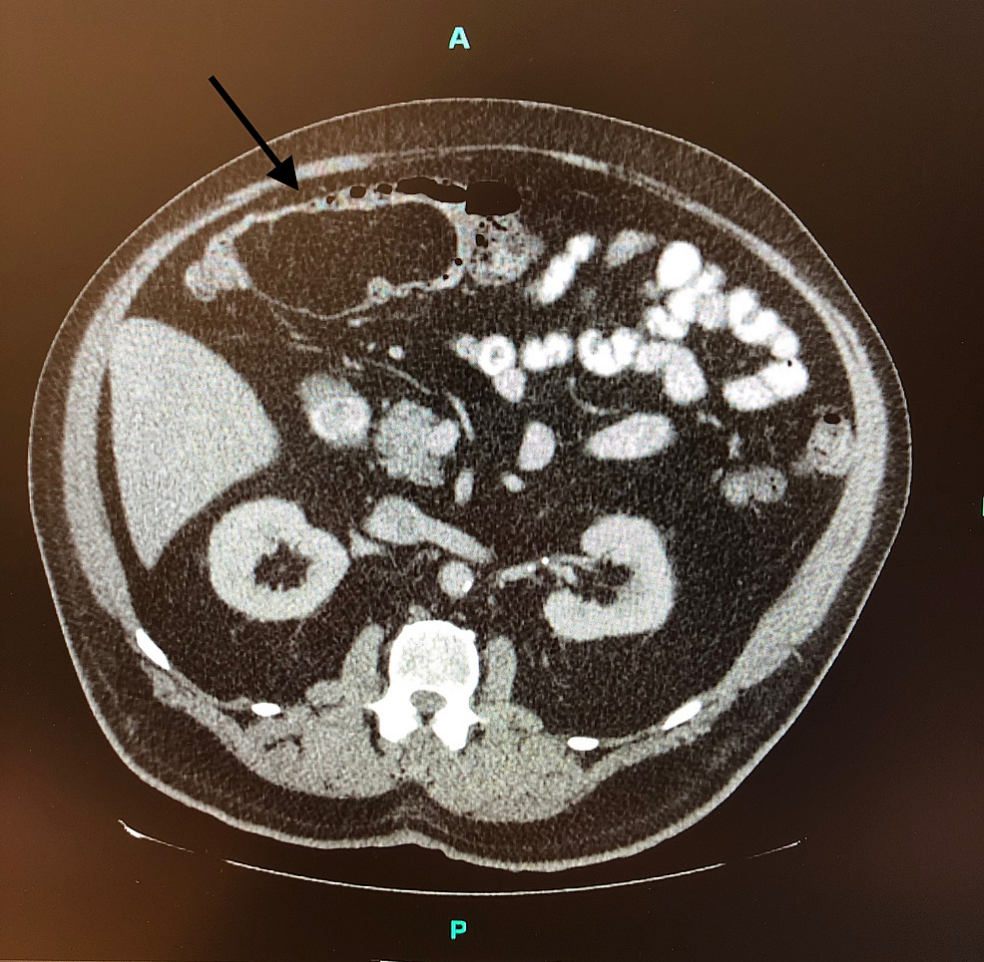
Cross-sectional image depicting a large, homogeneous, fatty density (black arrow) consistent with a lipoma causing a near-complete obstruction of the transverse colon

A 72-year-old male with a history significant for hypertension, hyperlipidemia, obesity, and peripheral vascular disease requiring lower extremity arterial bypass presented for surgical evaluation of a giant lipoma in the transverse colon. On his recent screening colonoscopy, the referring gastroenterologist had identified a 7 cm lipoma in the transverse colon. Previous colonoscopies had also revealed colonic lipomas, but they were noted to be small and clinically insignificant. Upon evaluation, the patient had no symptoms or complaints and reported regular bowel function. During the examination, his abdomen was soft, non-tender, and non-distended with no palpable masses appreciated. Cross-sectional imaging was obtained to further define the mass and demonstrated a homogenous fatty density nearly obstructing the colonic lumen (Figure 2).

Due to the significant size of the lipoma and concern for impending obstruction, the patient was taken for surgical intervention. With the negligible risk for malignancy, the performance of a formal oncologic resection was unnecessary. He underwent a successful, robotic-assisted, segmental transverse colectomy with the creation of a stapled, isoperistaltic, side-to-side colocolonic anastomosis. His recovery was complicated by postoperative ileus, which was managed conservatively with nasogastric tube decompression and supportive care. On postoperative Day 9, the patient had a return of bowel function and his diet was subsequently advanced in a step-wise fashion. He was discharged on postoperative Day 11, tolerating an oral diet with continued bowel function and adequate pain control with oral medications. The final pathological review of the specimen demonstrated a 10.5 cm submucosal lipoma with focal areas of intralesional necrosis but no evidence of changes in the overlying mucosa.

Case 3

**Figure 3:**
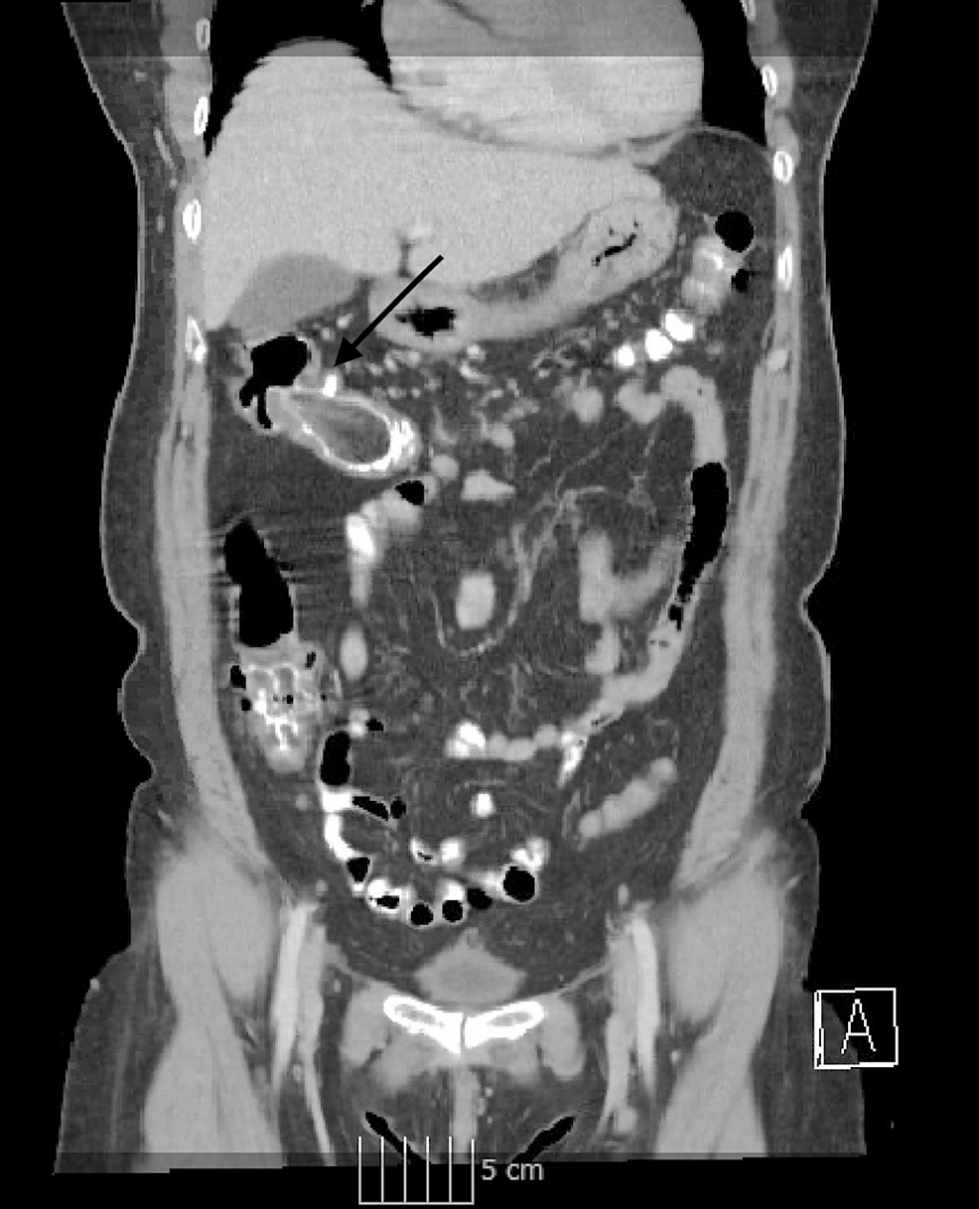
CT demonstrating a giant colonic lipoma with intussusception of the ascending colon (black arrow)

A 58-year-old female with a history significant for gastric ulcers presented to the emergency room for evaluation after three days of epigastric pain associated with dark, loose stools. A review of systems and laboratory workup revealed no significant findings. On examination, the patient’s abdomen was soft and non-distended with mild tenderness in the epigastrium and right upper quadrant. CT evaluation of the abdomen and pelvis (Figure 3) exhibited a 3.5 x 2.8 cm lipoma in the ascending colon with evidence of colocolonic intussusception and numerous prominent lymph nodes in the right abdomen measuring up to 0.8 cm. Of note, a previous colonoscopy had demonstrated a 2.5 cm pedunculated lipomatous polyp in the ascending colon, but the patient had remained asymptomatic with no previous evidence of obstructive potential on endoscopy.

The patient proceeded to the operating room and intussusception of the ascending colon was identified intraoperatively. Due to the patient’s emergent presentation and inability to confirm the diagnosis endoscopically, the decision was made to perform a formal oncological resection. A successful laparoscopic right hemicolectomy was performed with the creation of a stapled, isoperistaltic, side-to-side ileocolic anastomosis. On postoperative Day 5, the patient was discharged home following an uncomplicated recovery. Pathologic examination of the specimen confirmed a 4.3 x 2.9 x 2.8 cm pedunculated, lobulated, submucosal lipoma with congestion of the overlying mucosa. The neighboring colon exhibited evidence of colocolonic intussusception with vascular congestion, reactive changes, hemorrhage, and focal acute and chronic inflammation.

Case 4

**Figure 4:**
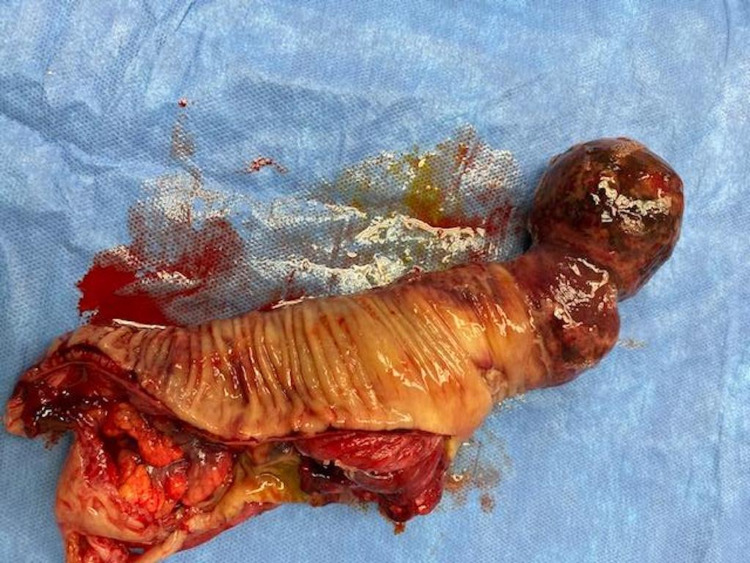
Surgical specimen consisting of a giant colonic lipoma and segment of intussuscepted colon

Boyack et al. previously reported a 33-year-old male who presented at our institution with two to three weeks of abdominal pain associated with vomiting and intermittent hematochezia and a palpable, mobile mass in the left lower abdomen who was found to have a 5 cm submucosal lipoma causing colocolonic intussusception of the sigmoid colon [6]. This patient was managed successfully with a laparoscopic, segmental colectomy of the descending colon and creation of a stapled, isoperistaltic side-to-side colocolonic anastomosis, with the resolution of symptomatology following surgical resection (Figure 4).

## Discussion

Colonic lipomas are of mesenchymal origin and are predominantly found in the right side of the colon in either the submucosal or subserosal spaces [1]. Previous reports have documented variability in sizes ranging from 0.35-10 cm in diameter, with only 30% reaching sizes greater than 2 cm [3]. As most lipomas remain asymptomatic, they are usually identified incidentally during surveillance colonoscopy, CT imaging, or autopsy. However, when symptoms develop, they are generally associated with lipomas greater than 2 cm in diameter, with giant lipomas posing the greatest risk for complications. Despite the relative rarity and mostly asymptomatic nature of colonic lipomas, their proper identification and diagnosis are vital to ensure correct management, as they can resemble gastrointestinal stromal tumors [12], harbor premalignant lesions (tubulovillous adenomas) [13], or assist in identifying genetic syndromes (Cowden) [14]. Although lipomas retain no malignant potential, at times, they can be difficult to discern from liposarcomas or other malignant lesions, especially in the setting of intussusception [5,9]. Colonic lipomas historically represented a diagnostic challenge, though improved capabilities of imaging modalities in conjunction with endoscopic examination have resulted in improved diagnostic accuracy and largely replaced previously utilized radiographic modalities. Accurate diagnosis is crucial in facilitating appropriate interventional consideration, both when identified incidentally or in the setting of a complication demanding more urgent intervention.

Intussusception represents one of the more serious complications of colonic lipomas and is defined as the invagination of a proximal portion of the intestine (intussusceptum) into a distal segment (intussuscipiens), leading to a telescoping of one segment into another. This pathology is of great concern, as it restricts perfusion to the involved segment of the bowel, which can result in ischemia, necrosis, or perforation. Colocolonic intussusception is regarded as especially concerning, as anatomical attachments of the colon are typically protective and approximately two-thirds of cases are resultant due to a primary malignancy [4]. With the risk of underlying malignancy and questionable integrity of the intussuscepted colon, surgical resection is historically regarded as the most appropriate intervention, as lesions capable of facilitating intussusception are generally too large for endoscopic resection, and endoscopy may result in perforation of the ischemic colon.

Surgical intervention has long been the standard of treatment for intussusception in adults. The traditional operation for colocolonic intussusception due to a giant colonic lipoma has been either a segmental colectomy or a formal hemicolectomy. These techniques enable adequate resection of the colon and associated lymphatic tissue while ensuring adequate margins are obtained when concerned for an underlying malignancy. As minimally invasive techniques have developed, the incorporation of laparoscopic and robotic approaches has become increasingly common, which is exemplified in our series of patients. However, some alternative approaches to consider include colotomy with enucleation and laparoscopic-assisted submucosal excision [15]. These approaches are typically more appropriate in the non-emergent setting for lesions not amenable to endoscopic resection or when colonic preservation is desired. When determining which surgical technique to employ, the decision should be individualized for each patient’s scenario while accounting for the risk of underlying malignancy.

Endoscopy remains the gold standard for the identification and diagnosis of colonic lipomas. Hallmark findings, such as the “tent sign,” “pillow sign,” and “naked fat sign” are diagnostic criteria for colonic lipomas [8,16]. However, the endoscopic treatment of lipomas was previously limited to symptomatic lesions with favorable morphological characteristics (pedunculated) and smaller than 2 cm in diameter. Though, as endoscopic instruments and techniques have improved, endoscopists have been increasingly able to resect colonic lipomas with a broader range of characteristics. Recently, a review by Bronswijk et al. evaluated the use of various endoscopic techniques in the treatment of 77 large, symptomatic colonic lipomas with a mean lesion size of 4.5 cm [17]. Techniques included: Unroofing, which involves snare removal of the overlying mucosa thus enabling spontaneous expulsion of the adipose tissue into the colonic lumen; Dissection-based techniques, which include endoscopic submucosal dissection and dissection of the lesion’s stalk; Endoscopic mucosal resection and loop-assisted-snare techniques, which involves the amputation of a lesion utilizing a loop and either hot or cold snare. Aside from the unroofing technique, all approaches demonstrated excellent results with resolution and clinical remission in greater than 93% of patients [17]. Despite excellent rates of resolution, endoscopic mucosal resection and loop-assisted-snare techniques also led to increased rates of adverse effects, including perforation. Thus, although advancements in endosurgical techniques have broadened the number of lipomas endoscopists can manage, it is accompanied by a real risk for complications that may require further urgent surgical intervention. This risk may be further compounded when utilizing endoscopic approaches to manage intussusception caused by giant lipomas. It may be more prudent for upfront surgical resection of larger lesions.

Aside from determining the best technique, defining when and which lipomas merit intervention has been a topic of debate. Historically, the intervention has been driven by symptomatology and the size of the lesion. However, as surgical techniques continue to be refined, a more proactive approach to resection would likely help minimize detrimental complications and emergent presentations. Traditionally, the intervention has been limited to symptomatic lipomas, typically with a diameter > 2 cm, as the slow-growing nature of lipomas meant small, asymptomatic lesions had a low likelihood of becoming clinically relevant [8,18]. However, as Siu et al. reported, initially irrelevant lipomas can retain the ability to grow and become symptomatic in a relatively short amount of time [19]. This finding was also reflected in our patients, as 50% had previous colonoscopies documenting small, asymptomatic lipomas. However, this growth pattern may not be applicable to all lesions, thus creating difficulty with predicting the risk of growth. Hence, a more assertive management approach should be considered. Resection of lipomas prior to them becoming symptomatic or developing complications such as obstruction, intussusception, or hemorrhage may be useful in preventing septic complications, extended hospitalizations, and decreasing associated hospital costs. Furthermore, more liberal use of surgical techniques should be considered as bowel-conserving approaches enable the removal of lesions without the loss of colonic length.

## Conclusions

In conclusion, we present a series of giant colonic lipomas managed safely and effectively with minimally invasive surgical resection. As our series and other reports have demonstrated, colonic lipomas may retain the ability to grow and become symptomatic over time. It may thus be pragmatic to consider resection of colonic lipomas prior to the development of symptoms or more emergent complications.
